# Effectiveness of unguided digital cognitive behavioral therapy for insomnia on depressive symptoms: a systematic review and meta-analysis of randomized controlled trials

**DOI:** 10.3389/fpsyt.2025.1718949

**Published:** 2026-01-12

**Authors:** Peixuan Zhong, Qian Zhu, Tiemei Li, Li Tong, Haijing Wang, Wen Peng

**Affiliations:** 1 Department of Traditional Chinese Medicine, Qinghai University Medical College, Xining, China; 2 Department of Maternal, Child and Adolescent Health, School of Public Health, Anhui Medical University, Hefei, China; 3 Qinghai Provincial Key Laboratory of Traditional Chinese Medicine Research for Glucolipid Metabolic Diseases, Xining, China; 4 Department of Public Health, Qinghai University Medical College, Xining, China

**Keywords:** cognitive behavioral therapy, dCBT-I, depressive, insomnia, meta-analysis

## Abstract

**Objective:**

This study aims to assess the intervention effects of unguided digital cognitive behavioral therapy for insomnia (dCBT-I) on patients with comorbid depression and insomnia through a systematic review and meta-analysis, in order to determine its effectiveness as a treatment strategy for comorbid depression and insomnia.

**Methods:**

We conducted a systematic search across PubMed, Cochrane, Embase, ClinicalTrials.gov, and PsycINFO.databases to identify randomized controlled trials (RCTs) focusing on adults with comorbid insomnia and depressive symptoms, excluding those with severe physical illnesses or psychiatric disorders. Following the removal of duplicates, 1842 articles were screened, resulting in the inclusion of 16 RCTs in the final meta-analysis.

**Results:**

Post-treatment evaluations revealed that digital cognitive behavioral therapy for insomnia (dCBT-I) demonstrated a significant impact on insomnia (SMD = -0.94; 95% CI: -1.40 to -0.48; *p* < 0.001; k = 16), though substantial heterogeneity was observed (I² = 98.63%; Q = 1250.89, df = 15, *p* < 0.05). For depressive symptoms, dCBT-I demonstrated a moderate effect (SMD = -0.63; 95% CI: -0.81 to -0.46; *p* < 0.05; k = 16), with high heterogeneity (I² = 81%; Q = 78.31, df = 15, *p* < 0.05).Strong outcomes were observed in both the Web and APP cohorts when analyzing program carriers, with notable findings in the moderately depressed subgroup.

**Conclusions:**

This meta-analysis evaluated the efficacy of therapist-unguided digital cognitive behavioral therapy (dCBT-I) for insomnia and depression, dCBT-I demonstrated significant reductions in insomnia severity and depressive symptoms within the studied population, despite substantial heterogeneity. It also demonstrated moderate to large effects for moderate-to-severe depression, further supporting the effectiveness of unguided dCBT-I.

**Systematic Review Registration:**

https://www.crd.york.ac.uk/PROSPERO/view/CRD420251044042, identifier CRD420251044042.

## 1 Introduction

Insomnia, as one of the most common sleep disorders, is associated with the occurrence and development of many diseases. Among the general adult population, approximately 10% to 50% have experienced short-term or long-term insomnia (1). According to the Diagnostic and Statistical Manual of Mental Disorders-V (DSM-V) diagnostic criteria, insomnia disorder is defined as subjective reports of difficulty falling asleep, sleep maintenance disorders, or inability to achieve restorative sleep quality, and the sleep difficulty occurs at least 3 nights per week (2). Insomnia is also associated with daytime dysfunction such as fatigue, depression, anxiety, irritability, and decreased cognitive function (3).

Epidemiological investigations have shown that there is a relatively high incidence of comorbidity between insomnia and depression, and possess a degree of correlation and bidirectionality (4). Among patients meeting the diagnostic criteria for insomnia, the prevalence of depression is significantly higher than that in patients without insomnia (5).Over 90% of individuals with depression experience a decline in sleep quality. Insomnia is a prevalent residual symptom of depression, and individuals with depression who have persistent insomnia face a notably higher risk of relapse (6, 7). The prevalence of comorbid insomnia and depression has emerged as a critical concern in clinical psychology (8). These two mental health conditions perpetuate a mutually reinforcing pathological cycle through complex mechanisms involving neural circuitry, endocrine function, inflammatory processes, and structural brain changes (9, 10). This interplay significantly undermines individual mental resilience and social adaptability, presenting a crucial public health challenge in the 21st century that requires urgent attention and intervention (11).

In clinical settings, insomnia symptoms are typically addressed through pharmacotherapy (12). However, hypnotics inadequately mitigate depressive comorbidities and often result in significant daytime side effects (13). Cognitive Behavioral Therapy for Insomnia (CBT-I) is recognized as the primary treatment recommended by the American Academy of Sleep Medicine (AASM, 2021) due to its comprehensive intervention benefits (14). Through the implementation of evidence-based strategies—including sleep restriction, stimulus control, and cognitive restructuring—this therapy enhances sleep efficiency, rectifies dysfunctional sleep-related cognitions, and synergistically regulates the central nervous system mechanisms underlying the sleep-wake rhythm (15). Clinical studies have demonstrated that cognitive-behavioral therapy for insomnia (CBT-I) significantly ameliorated insomnia symptoms and markedly reduced depression scale scores in patients with comorbid depression (16–18).

Although the efficacy of traditional cognitive behavioral therapy for insomnia (CBT-I) in treating insomnia and depression has been confirmed, it also has some limitations, such as a shortage of therapist resources, high treatment costs, and restrictions in terms of time and location (19, 20). With the development of technology, digital cognitive behavioral therapy for insomnia (dCBT-I) has been developed and studied in the past decade (21). This technology is relevant to various vehicles, including computers, the Internet, and smartphone applications. Programs for dCBT-I encompass not just the essential elements of conventional CBT-I, but also offer services tailored to individual needs (22–24).

A portion of dCBT-I includes a human feedback component that gives personalized advice based on what the participant has completed and is supervised to increase the participant’s compliance (25). There are also unguided dCBT-I programs that are algorithmically based to provide feedback from a virtual therapist without any human involvement component (26, 27). In groups that show greater prejudice against mental health problems, there is a clear preference for self-help psychological interventions and an increasing acceptance of low-intensity, easily accessible treatments (28, 29).

There are subgroup analyses of fully automated dCBT-I in previous studies that have shown small to moderate therapeutic effects on depression, but these studies did not differentiate between populations and included populations with other co-morbidities (such as cancer or pain), which may have influenced the results of the study (30). Therefore, the effects of dCBT-I on insomnia and depression need to be further investigated in a generalized population without the involvement of a therapist. This meta-analysis was intended to evaluate the efficacy of unguided digital cognitive behavioral therapy for insomnia (dCBT-I) for the treatment of insomnia and depressive symptoms in the adults with comorbid insomnia and depressive symptoms, excluding those with severe physical illnesses or psychiatric disorders, with subgroup analyses of dCBT for both web and app vehicles, and a study of patients with moderate and higher levels of depression to evaluate the efficacy of dCBT-I.

## 2 Method

### 2.1 Data sources and search strategies

This work follows the Cochrane Handbook for Systematic Reviews of Interventions and the Preferred Reporting Items for Systematic Reviews and Meta-Analyses (PRISMA) (31, 32). The study protocol was prospectively registered with the International Prospective Register of Systematic Reviews (PROSPERO) under registration number CRD420251044042, with no prior publication of the protocol. The RCTs reporting depression outcomes in adult insomnia patients were searched in the PubMed, Cochrane, Embase, ClinicalTrials.gov, and PsycINFO databases up to May 25, 2025. These trials compared unguided digital cognitive behavioral therapy for insomnia (dCBT-I) against placebo or standard care. Further studies or follow-up studies from the original trials were also eligible if they met the predefined inclusion criteria. The search query was applied in PubMed as follows: (“sleep initiation and maintenance disorders”[MeSH Terms] OR “insomnia”[Title/Abstract] OR “early awakening” [Title/Abstract] OR “sleeplessness”[Title/Abstract] OR “sleep initiation dysfunction”[Title/Abstract] OR “DIMS”[Title/Abstract] OR “sleep”[Title/Abstract]) AND (“depressive disorder”[MeSH Terms] OR “melancholia”[All Fields] OR “endogenous depression”[All Fields] OR “depression unipolar”[All Fields] OR “neurotic depression”[All Fields] OR “psychological”[All Fields] OR “depression”[All Fields]) AND (“CBT”[Title/Abstract] OR “cognitive behavioral therapy”[Title/Abstract] OR “cognitive therapy”[Title/Abstract] OR “behavioral therapy”[Title/Abstract]) AND (“digital”[Title/Abstract] OR “internet”[Title/Abstract] OR “online”[Title/Abstract] OR “web”[Title/Abstract] OR “telephone”[Title/Abstract] OR “mobile”[Title/Abstract] OR “app”[Title/Abstract] OR “application”[Title/Abstract] OR “smartphone”[Title/Abstract] OR “computer”[Title/Abstract] OR “computerized”[Title/Abstract] OR “ehealth”[Title/Abstract] OR “AI”[Title/Abstract]).

### 2.2 Inclusion and exclusion criteria

The inclusion criteria followed the Participant, Intervention, Comparison, Outcome, and Study (PICOS) guidelines. Studies meeting the following criteria were included in this meta-analysis: (1) Participants: The study population consisted of adult patients ≥18 years of age, with clinician-diagnosed or symptomatic insomnia (per DSM/ICSD criteria), regardless of gender/education (2, 33, 34); (2) Intervention: Unguided digital CBT-I (computer/internet/apps) incorporating ≥1 cognitive (e.g., cognitive restructuring) and ≥1 behavioral component (e.g., stimulus control/sleep restriction); (3) Comparison: Placebo, active control, waitlist, or standard care; (4) Outcomes: Baseline and endpoint depressive symptoms measured by validated scales (e.g., HAMD, PHQ-9); studies including anxiety comorbidities were not excluded; (5) Study design: Randomized controlled trials (RCTs).

The following exclusion criteria were used: (1) Serious physical illness (e.g., cancer, Parkinson’s), substance abuse, organic/primary sleep disorders (e.g., sleep apnea, RLS), psychiatric disorders (e.g., bipolar disorder, schizophrenia), neurological disorders, acute suicide risk, severe cognitive impairment, or pregnancy; (2) Non-dCBT-I therapies (e.g., pharmacotherapy alone), unstandardized dCBT-I, or combined interventions without isolated dCBT-I effect analysis; (3) Incomplete outcome reporting (e.g., single-scale data) or irretrievable data; (4) Non-RCTs, case reports, protocols, reviews, duplicate publications, or conference abstracts (incomplete methods); (5) The cognitive behavioral therapy for depression (CBT-D).

### 2.3 Data extraction and quality assessment

Two researchers (PXZ, QZ) independently extracted and reviewed studies for consideration based on the eligibility criteria. If there was disagreement, the decision was reviewed by a third researcher (HJW). Any discrepancies were resolved through discussion between all authors. Duplicate articles were removed; titles and abstracts of potential studies were screened; and full texts that met the criteria were further evaluated. We identified publications of the same study so that each study, rather than each report, was the unit of analysis in the review. From the included studies, we extracted: sample size, mean age, and control group. Detailed information, including title, authors, year of publication, study design, number of dCBT-I sessions, and treatment duration, was extracted. In addition, relevant study variables were assessed using an assessment tool, and pre- and post scores were extracted for the intervention and control groups. Two reviewers independently assessed the methodological quality of the included RCTs using the Cochrane Risk of Bias Assessment Tool (RoB 2.0) (35). The assessment included randomization sequence generation, allocation concealment, participant and investigator blinding, completeness of outcome data, selective outcome reporting, and other biases. The results showed that of the 16 studies, all 16 had a low risk of bias in terms of “Incomplete outcome data” and “Selective reporting”. Regarding other bias types, 1 study was judged to have an unclear risk of bias in “Random sequence generation” due to “not mentioned”, 2 studies were judged to have an unclear risk of bias in “Allocation concealment” due to “not mentioned”, 3 studies were judged to have an unclear risk of bias in “Blinding of participants and personnel” due to “not mentioned”, 3 studies were judged to have an unclear risk of bias in “Blinding of outcome assessment” due to “not mentioned”, and 2 studies were judged to have an unclear risk of bias in “Other bias” due to “not mentioned”. No studies were found to have a high overall risk of bias.

### 2.4 Statistical analysis

Standardized Mean Difference (SMD) was used as an indicator of the combined effect sizes of the continuous variables, and the overall between-group SMD was calculated based on the difference in post-intervention outcome measures between the dCBT-I intervention and control groups, with 95% confidence intervals (CI) reported. If the study did not provide the relevant data, they were obtained by graphical extraction or by contacting the original authors for additional information. Consistent with prior research, analyses were conducted to compare post-treatment values between groups (30).

Heterogeneity was assessed via the Cochrane Q test and the I² statistic.I² values ranging from 0% to 100%, with larger values indicating higher heterogeneity. Generally, I² ≤ 25% is considered low heterogeneity, 25%≤ I² ≤ 50% as moderate heterogeneity, and I² > 50% as substantial heterogeneity. The choice of meta-analytic model was based on the degree of statistical heterogeneity. A random-effects model was used if I² > 50% or if the p-value for Cochran’s Q test was < 0.10, otherwise, a fixed-effects model was applied. Publication bias was assessed using Egger’s test. Possible reporting bias and small study effects were assessed using contour-enhanced funnel plots.

### 2.5 Subgroup analysis

The effects of the interventions on the combined outcomes were analyzed by grouping the studies according to the type of intervention (different dCBT-I vectors) included in the study, comparing differences in effect sizes between subgroups. And the effect of dCBT-I on different levels of depression was assessed by grouping them according to the level of depression at baseline. We also conducted a separate analysis of studies with moderate to severe depression levels. Subgroup analyses also used a random effects model to calculate the combined SMD and 95% CI for each subgroup, and to determine the effect of grouping factors on outcomes by comparing the differences in effect sizes and statistical significance between subgroups. Statistical differences between subgroups were assessed at α = 0.05 significance level. All analyses were performed in Review Manager 5.4 and Stata 17.0.

## 3 Results

### 3.1 Study flow

**Figure 1:**
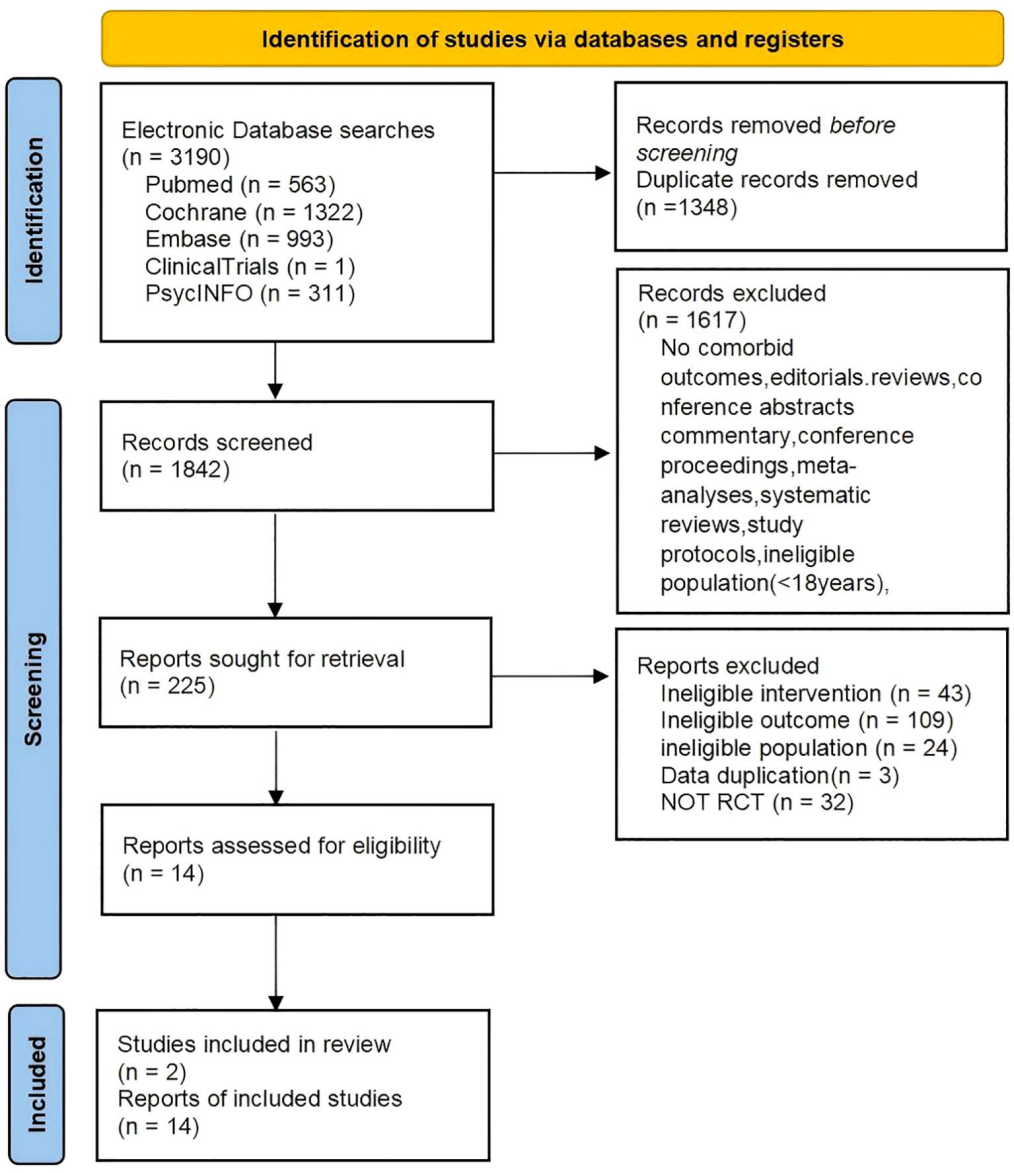
PRISMA flow diagram of study selection.

The flow of study selection is presented in the PRISMA flow diagram (**Figure 1**). A total of 1842 articles were identified after duplicate removal, of which 225 articles were assessed for full-text review. A final sample of 16 RCTs was included in the meta-analysis.

### 3.2 Study characteristics

**Table 1** describes the methodological and demographic characteristics of the 16 included studies. A total of 7488 participants were involved in the meta-analysis, with 3803 individuals allocated to the dCBT-I group. The median sample size of study was 100 participants (range: 46 to 3352). The mean age of the dCBT-I group and the control group was 36.40 ± 14.96 and 36.30 ± 14.94 years, respectively. Subjects in the control group received either active interventions, including sleep education or general health education (not sleep-specific), or passive controls, including routine treatment and waiting lists. All studies used a parallel design, with 15 studies using a two-arm trial and one using a three-arm trial, but one of the three-arm studies did not meet the inclusion criteria, so only two of the data sets were included. For the treatment group, dCBT-I treatment sessions ranged from 5 to 18, with a mean of 7.31 sessions for the included studies. These studies were conducted in Australia (n=3), China (n=2), Germany (n=3), Iranian (n=1), the Netherlands (n=2), Switzerland (n=1), the United Kingdom (n=2), and the United States (n=2).

**Table 1:** Summary characteristics of the included studies.

NO.	Author	Year	Title	Country	Total sample size (% Female)	Mean age, year (SD)	Study design	dCBTi components	Intervention type	No. of sessions	Time point of post-assessment	Insomnia outcome measurement	Depression outcome measurement
1	Lancee et al.2012 (36)	2012	Internet-delivered or mailed self-help treatment for insomnia? A randomized waiting-list controlled trial	Netherlands	623 (69.7)	ELE: 52.2 (11.4) PP: 51.2 (12.8) WL: 51.9 (12.2)	3 parallel arms (ELE, PP, WL)	PE,SC,SH,PMR,SR,CR	Web	6	6 weeks	SLEEP-50	CES-D
2	Christensen et al.2016 (37)	2016	Effectiveness of an online insomnia program (SHUTi) for prevention of depressive episodes (the GoodNight Study): a randomised controlled trial	Australia	1149 (73.5)	SHUTi: 42.49 (12.17) Health Watch: 42.51 (12.24)	2 parallel arms (SHUTi, HealthWatch)	SR,SC,CR,SH,RP	SHUTi (Web)	6	6 weeks	ISI	PHQ-9
3	Horsch et al.2017 (38)	2017	Mobile Phone-Delivered Cognitive Behavioral Therapy for Insomnia: A Randomized Waitlist Controlled Trial	Netherlands	151 (62.3)	CBT-I: 39.0 (13.0) WL: 41.0 (13.9)	2 parallel arms (CBT-I, WL)	SD,RE,SR,SH,CT	Sleepcare App	4	6–7 weeks	ISI	CES-D
4	Glozier et al.2019 (39)	2019	Adjunctive Internet-delivered cognitive behavioural therapy for insomnia in men with depression: A randomised controlled trial	Australia	87 (0.0)	CBT-I: 58.6 (6.3) PE: 58.1 (6.1)	2 parallel arms (CBT-I,PE)	SC,SH,CR,RP	SHUTi (Web)	6	12 weeks	ISI	CES-D
5	Lorenz et al.2019 (40)	2019	Randomized Controlled Trial to Test the Efficacy of an Unguided Online Intervention with Automated Feedback for the Treatment of Insomnia	Switzerland	56 (69.6)	CBT-I: 41.72 (17.31) WL: 44.04 (20.05)	2 parallel arms (CBT-I,WL)	PE,SR,RE,SH,CR	Mementors-omnium (Web)	6	6 weeks	ISI	BDI-II
6	Henry et al.2020 (41)	2020	Insomnia as a mediating therapeutic target for depressive symptoms: A sub‐analysis of participant data from two large randomized controlled trials of a digital sleep intervention	UK	3352 (76.0)	Intervention: 29.6 (13.0) Control: 29.4 (12.9)	2 parallel arms (Intervention, Control)	SR, SC, RE, CR, PI, SH	Sleepio (Web)	6	8 weeks	SCI^*^	PHQ-9
7	Majd et al.2020 (42)	2020	Efficacy of a Theory-Based Cognitive Behavioral Technique App-Based Intervention for Patients With Insomnia: randomized Controlled Trial	Iranian	312 (55.7)	CBT-I: 36.21 (5.81) PE: 35.29 (5.76)	2 parallel arms (CBT-I, PE)	CBT(SR,SC,SH,RE,CT), TPB,HAPA	BCT App	6	6 weeks	ISI	HADS
8	Cheng et al.2020 (23)	2020	Efficacy of digital CBT for insomnia to reduce depression across demographic groups: a randomized trial	USA	1385 (78.8)	CBT-i: 44.5 (15.8) SHE: 45.7 (15.1)	2 parallel arms (CBTi, SHE)	SR,SC,CR,PI,RE,SH	Sleepio (Web)	6	12 weeks	ISI	QIDS
9	Chan et al.2021 (43)	2021	Treating depression with a smartphone-delivered self-help cognitive behavioral therapy for insomnia	China	320 (73.0)	proACT-S: 27.28 (7.25) WL: 27.26 (7.22)	2 parallel arms (proACT-S, WL)	SR,SC,ST,SH,RP	proACT-S App	6	6 weeks	ISI	CES-D
10	Zhang et al.2023 (26)	2023	Digital Cognitive Behavioral Therapy for Insomnia Using a Smartphone Application in China	China	82 (74.4)	dCBT-I: 49.6 (13.0) SE: 50.6 (14.1)	2 parallel arms (DCBT-I, SE)	SR,RT,SC,SH,CT	Resleep App	7	6 weeks	ISI	PHQ-9
11	Specht et al.2024 (27)	2024	Effectiveness and safety of an interactive internet-based intervention to improve insomnia: Results from a randomised controlled trial	Germany	290 (73.8)	SOM: 49.90 (14.67) TAU: 49.75 (13.55)	2 parallel arms (SOM, TAU)	PE,SR,SH,RA,RE,CT,RR	Somnovia (Web)	9	12 weeks	ISI	PHQ-9
12	Rötger et al.2024 (44)	2024	The clinical effect of digital cognitive behavioural therapy for insomnia in subgroups with depressive and anxiety symptoms: A secondary analysis of a randomized–controlled trial	Germany	88 (73.9)	dCBT-I: 42.98 (14.67) WLC: 41.63 (14.08)	2 parallel arms (dCBT-I, WLC)	SH,SR,SC,RT,CT	Somnio App	10	8 weeks	ISI	ADS-K
13	Sweetman et al.2024 (45)	2024	Digital cognitive behavioural therapy for insomnia versus digital sleep education control in an Australian community-based sample: a randomised controlled trial	Australia	62 (82.2)	CBTi: 52.82 (16.37) SE: 52.11 (16.41)	2 parallel arms (CBTi, SE)	PE,SC,SR,RT,RP,CR	Web	5	8 weeks	ISI	PHQ-9
14	Tamm et al.2025 (46)	2025	Emotional Processing Following Digital Cognitive Behavioral Therapy for Insomnia in People with Depressive Symptoms: A Randomized Clinical Trial	UK	205 (80.8)	CBTi: 49.7 (10.2) SHE: 48.8 (10.1)	2 parallel arms (CBTi, SHE)	SH,PR,SC,SR,CR,AT,IMA,MIN,PI	Sleepio (Web)	6	10 weeks	ISI	PHQ-9
15	Staiano et al.2025 (47)	2025	Assessment of an App-Based Sleep Program to Improve Sleep Outcomes in a Clinical Insomnia Population: randomized Controlled Trial	USA	132 (52.3)	SI: 37.3 (11.5) WL: 37.0 (9.6)	2 parallel arms (SI, WL)	MIN, CT,RE,SC,SH	Headspace App	18	3 weeks	ISI	PHQ-8
16	Schuffelen et al., 2024 (48)	2025	Digital CBT-I in Comorbid Insomnia and Depression: Clinical Outcomes From a Pragmatic Randomized Controlled Trial	Germany	140 (85.7)	dCBT-I: 39.56 (11.31) WL: 39.96 (12.07)	2 parallel arms (dCBT-I, WL)	PE,RT,SC,RE,CT	Somnio App	10	12 weeks	ISI	PHQ-9

ADS-K, Allgemeine Depressions-skala-Kruzform; AT, Autogenic training; BDI-II, Beck depression inventory, second edition; CES-D, Center for Epidemiological Studies Depression Scale; CBT, Cognitive behavioral therapy; CR, Cognitive restructuring; CT, Cognitive techniques; HADS, Hospital Anxiety and Depression Scale; HAPA, Health Action Process Approach; IMA, Imaginary; ISI, Insomnia severity index; MIN, Mindfulness and/or mediation; PE, Psycho-education; PHQ-9, Patient Health Questionnaire-9; PHQ-8, Patient Health Questionnaire-8; PI, Paradoxical intention; PP, Paper-and-pencil; PR, Progressive relaxation; PS, Psychoeducation; PMR, Progressive muscle relaxation; QIDS, Quick Inventory of Depression symptomatology - self Report; RA, Reduction of alcohol use at night; RE, Relaxationt echnigues, such as progressive musce relaxation; RP, Relapse prevention; RR, Reducing rumination; RT, Relaxation techniques; SC, Stimulus control; SD, sleep diary; SCI, Sleep Condition indicator,*Higher score indicates less impairment; SH, Sleep hygiene education; SOM, Somnovia; SR, Sleep restriction therapy; ST, Stress management; SHUTi, Sleep Health Utilization Tool – insomnia; TAU, Treatment as usual; TPB, Theory of Planned Behavior; WL, Wait-list.

### 3.3 Risk and bias assessment

**Figure 2:**
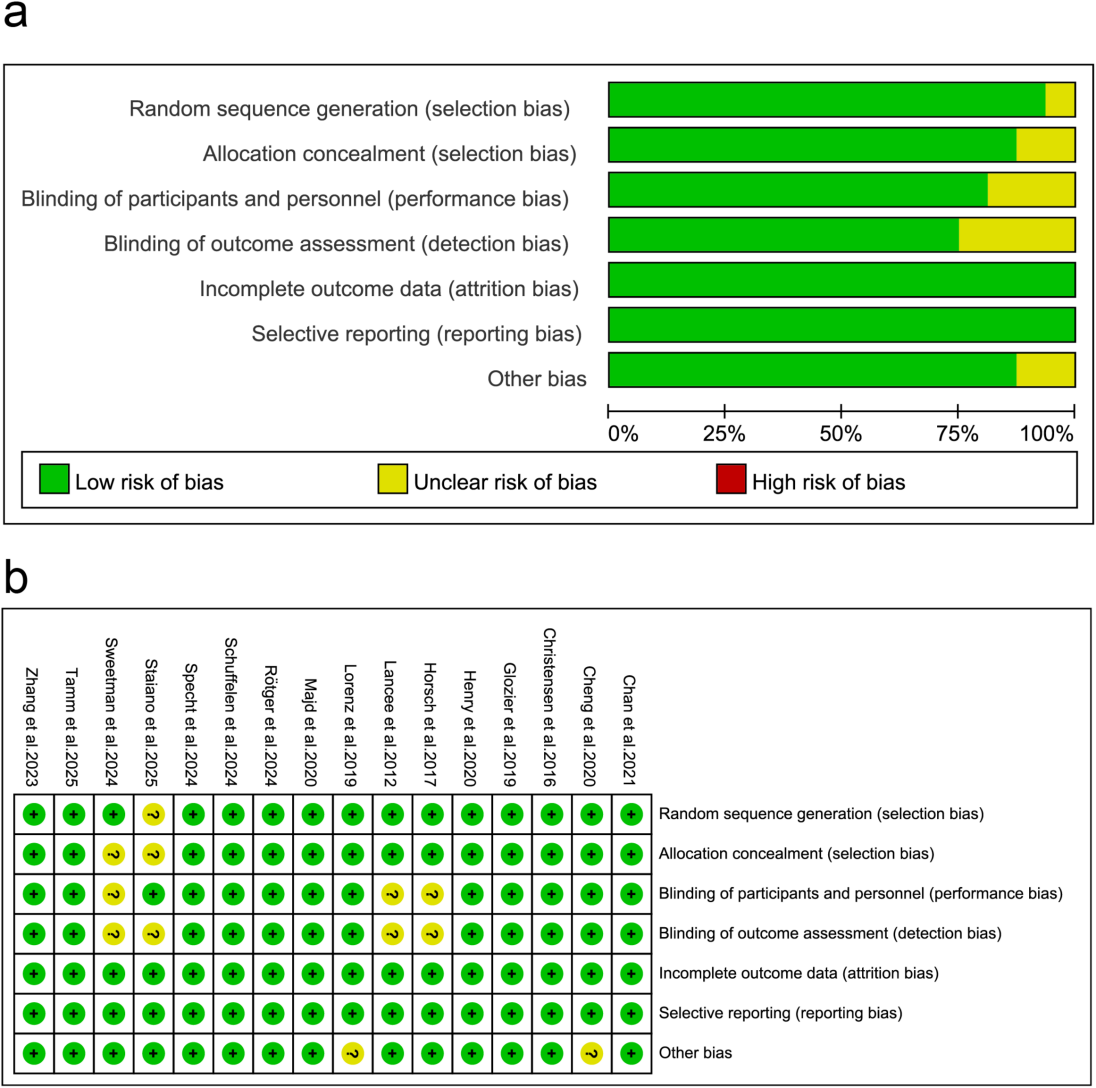
Risk of bias assessment.

The risk of bias for the included studies was assessed using the Cochrane Risk of Bias Tool (version 226), and the results are presented in **Figure 2**. Ten studies had an overall low risk of bias, six had a moderate risk of bias. Significant risk of bias was detected for measures from the outcome domains, mainly due to blinding of study participants and researchers, which may also lead to deviation from the intended intervention. The included studies were generally of high methodological quality, with a low overall risk of bias.

### 3.4 Treatment effects

#### 3.4.1 Sleep measures

Sleep outcome measures varied from study to study and included the Insomnia Severity Index (ISI), the Sleep Condition Index (SCI), and the Sleep-50 measure. The ISI was chosen as the primary sleep outcome measure. All studies used the ISI as an outcome of insomnia severity, except for two studies, which were Lancee et al., 2012 (49) and Henry et al., 2020 (40), one study used the SCI, and the other used the SLEEP-50 measure.

**Figure 3:**
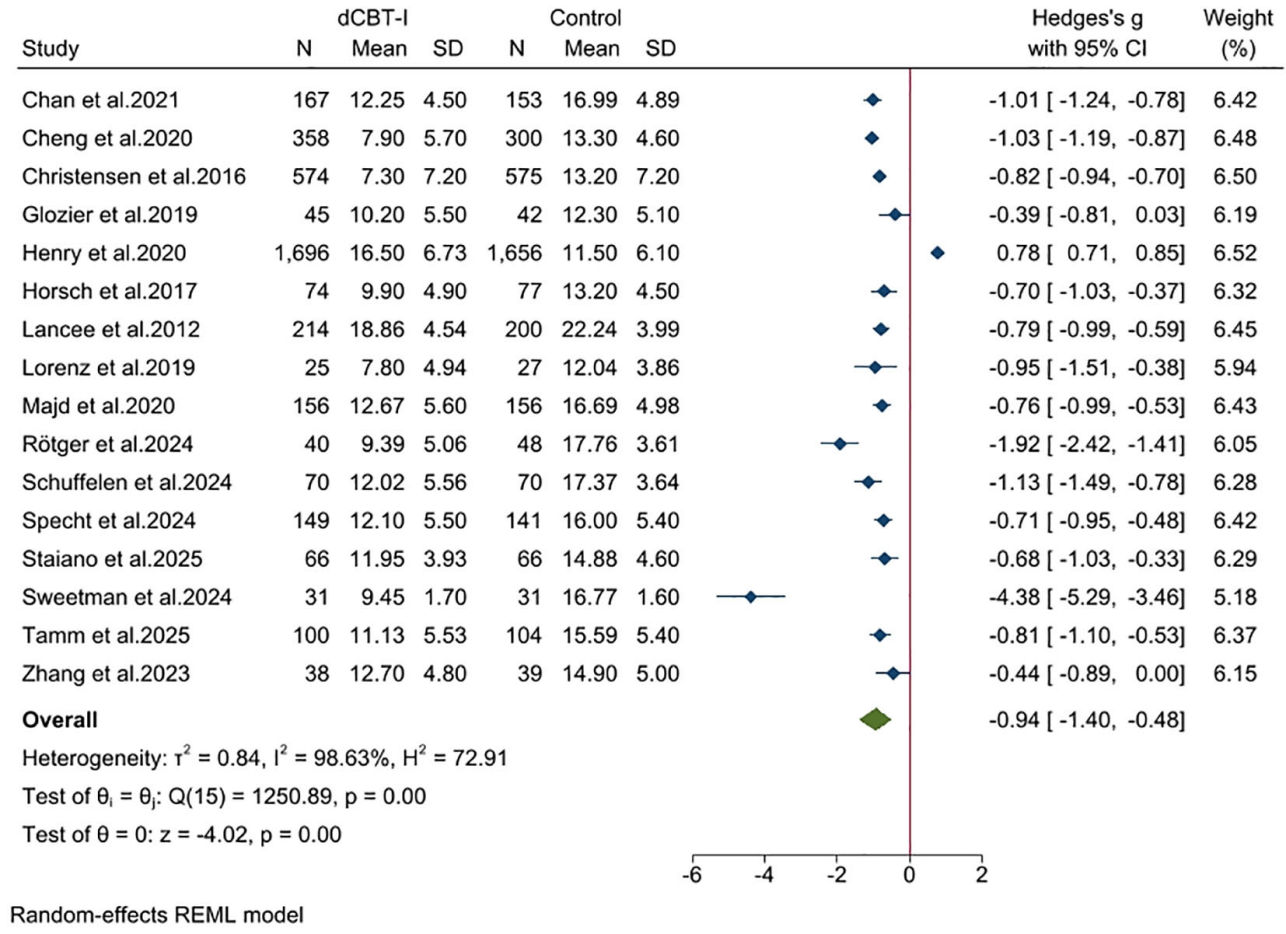
Effects of dCBT-I on sleep outcomes and sleep efficiency.

For the severity of insomnia after treatment, we found a large effect (**Figure 3**; SMD = -0.94; 95% confidence interval (CI): -1.40, -0.48; *p* < 0.001; k = 16). Statistical heterogeneity of effect sizes between studies was very high (I^2^ = 98.63; Q = 1250.89; df = 15; *p* < 0.05). In studies that included only ISI, we found that dCBT-I still had a substantial advantage (**Supplementary Figure 1**, SMD = -1.07; 95% CI: -1.51, -0.63; *p* < 0.05; I^2^ = 97.33; k = 14).

**Figure 4:**
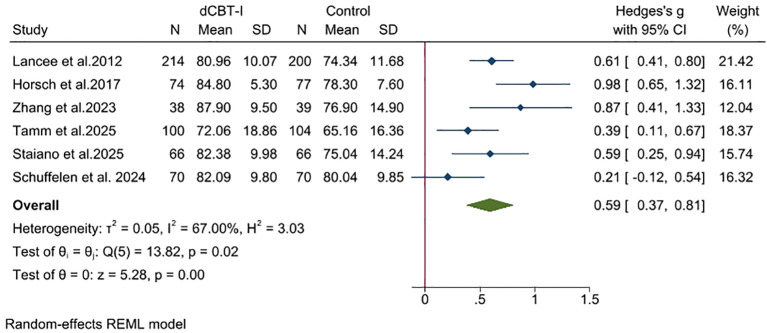
Effect of dCBT-I on sleep efficiency.

For sleep diary outcomes, the effect was significant with a moderate to large effect size for SE (**Figure 4**; SMD = 0.59; 95% CI: 0.37, 0.81; *p* < 0.05; I^2^ = 67; k = 6), SOL (**Supplementary Figure 2**, SMD = −0.42; 95% CI: −0.54 −0.30; *p* < 0.05; I^2^ = 3.05; k = 6) and WASO (**Supplementary Figure 3**; SMD = −0.49; 95% CI: −0.85, −0.13; *p* < 0.05; I^2^ = 84.68; k = 5), while the effect was significant with a small effect size for TST (**Supplementary Figure 4**; SMD = 0.28; 95% CI: 0.16, 0.40; *p* < 0.05; I^2^ = 1.12; k = 6).

#### 3.4.2 Depression measures

**Figure 5:**
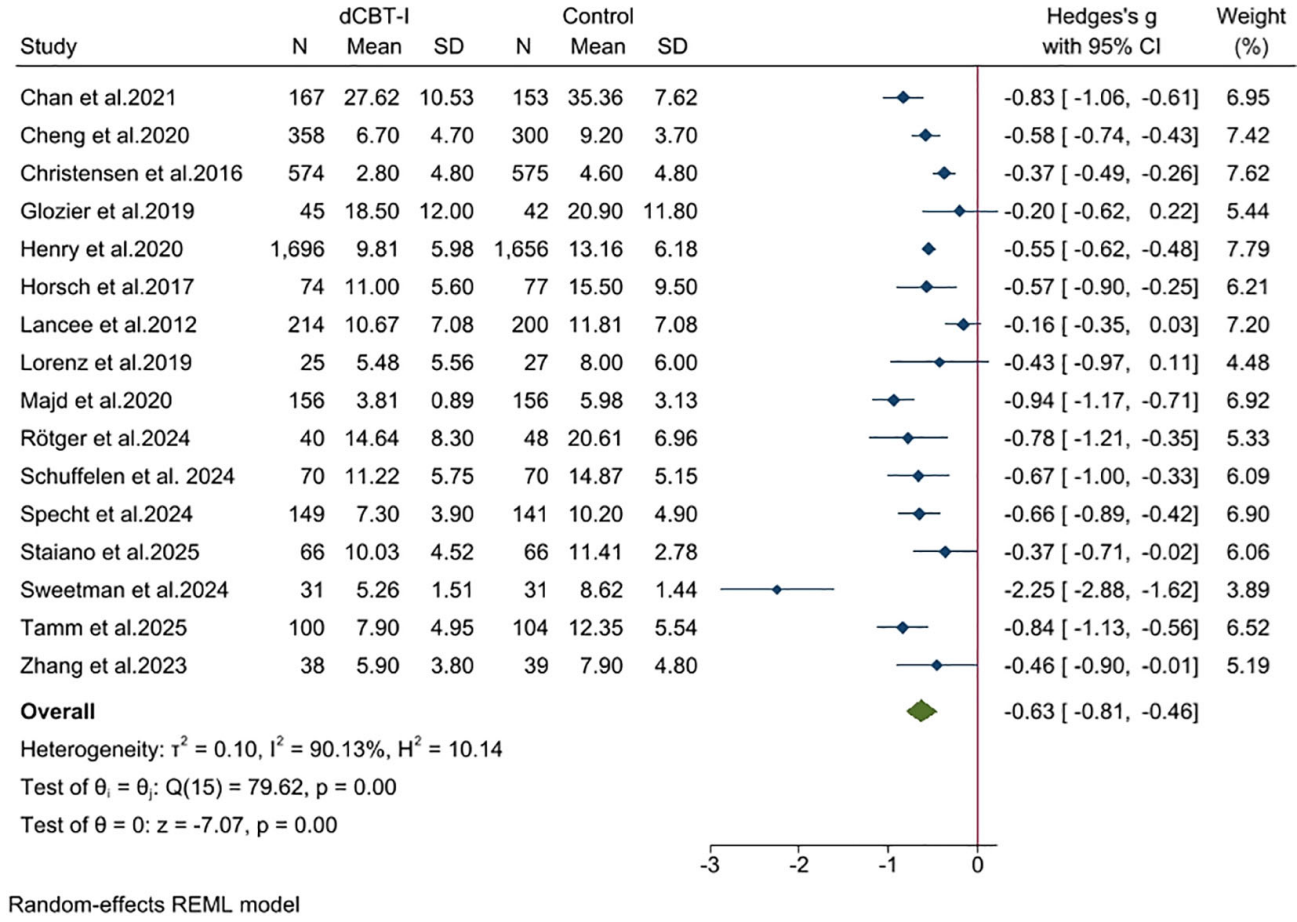
Effects of dCBT-I on depression.

All 16 studies reported the severity of depressive symptoms. The result measurement of depressive symptoms varies in different studies. Including the Center for Epidemiologic Studies Depression Scale (CES-D) and Patient Health Questionnaire-9 (PHQ-9), Allgemeiner Depressions-Skala (ADS-K), Hospital Anxiety and Depression Scale-Depression (HADS-D), Beck Depression Inventory (BDI-II), and Quick Inventory of Depressive Symptomatology (QIDS). In the post-treatment assessment, we found that digital cognitive behavioral therapy (dCBT-I) produced a moderate positive effect in treating depression. (**Figure 5**; SMD = -0.63; 95% CI: -0.81, -0.46; *p* < 0.05; k = 16). The statistical heterogeneity in effect sizes among studies was high (I^2^ = 81; Q = 78.31; df = 15; *p* < 0.05).

### 3.5 Subgroup analysis

**Figure 6:**
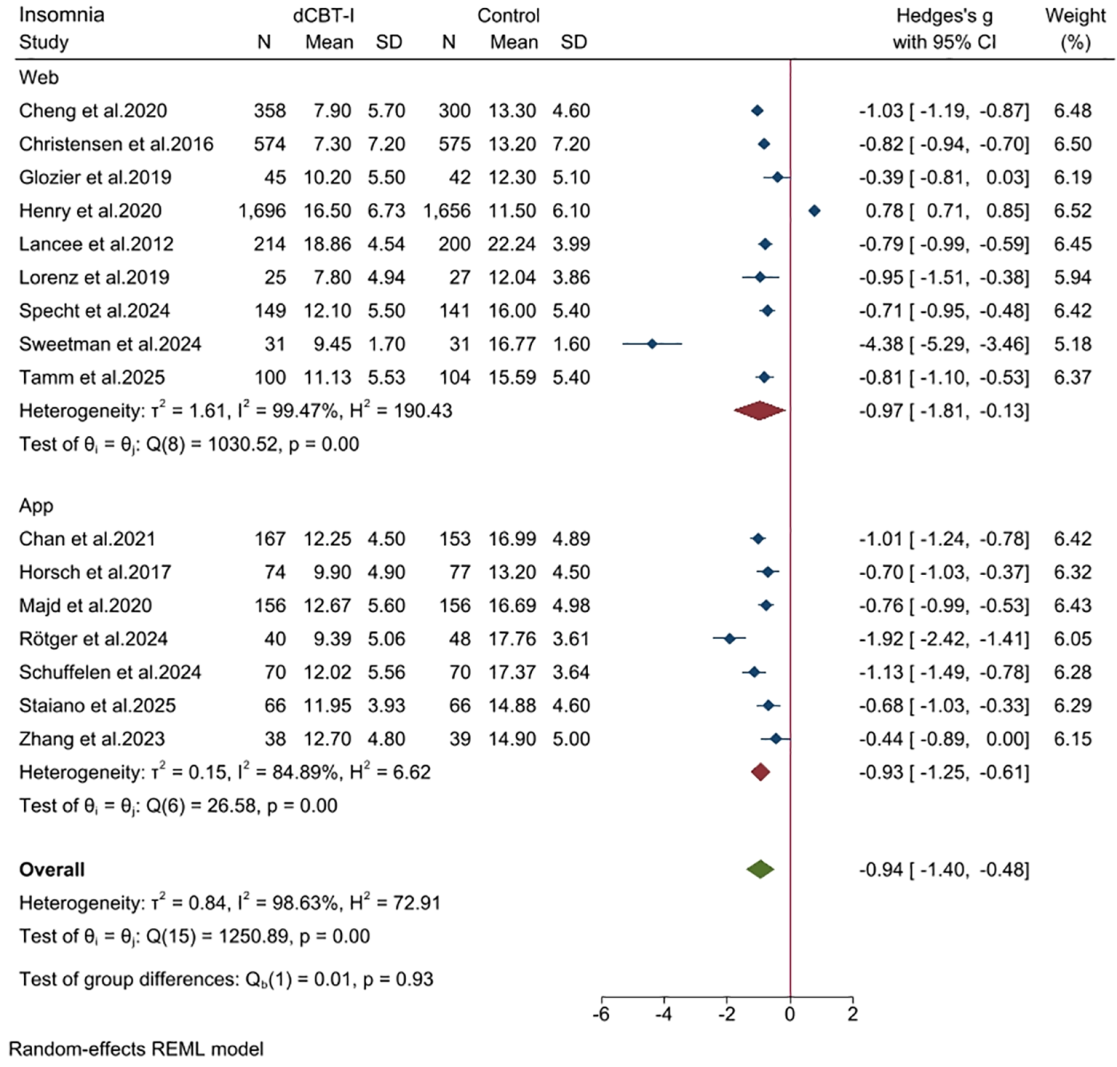
Subgroup analysis (Insomnia): Web vs. App.

**Figure 7:**
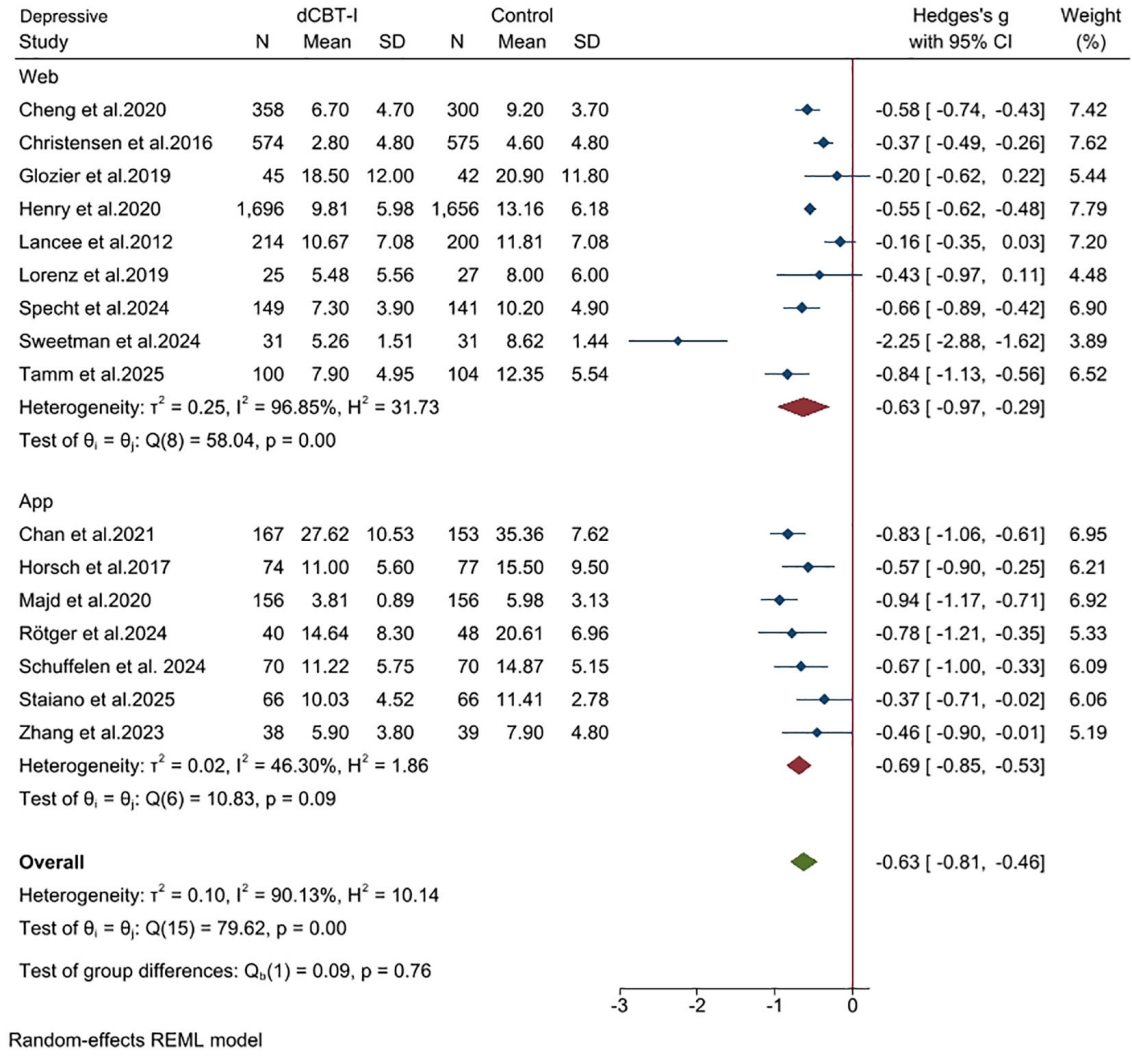
Subgroup analysis (Depression): Web vs. App.

An additional subgroup analysis was performed on 16 studies that reported relevant applications of the dCBT-I session. To compare the effects of different applied treatments, we categorized the 16 studies into two groups: (1) the web platform group, dCBT-I completers who applied the online web platform; and (2) the app group, dCBT-I completers who applied the mobile app. The treatment effect of the web group was significant for insomnia (**Figure 6;** SMD = -0.97; 95% CI: -1.18, -0.13; *p* < 0.05; I^2^ = 99.47; k = 9) and medium for depression (**Figure 7**; SMD = -0.63; 95% CI: -0.97, -0.29; *p* < 0.05; I^2^ = 96.85; k = 9). The treatment effect of the app group was also significant for insomnia (SMD = -0.93; 95% CI: -1.25, -0.61; *p* < 0.05; I^2^ = 84.89; k = 7), and medium for depression (SMD = -0.69; 95% CI: -0.85, -0.53; *p* < 0.05; I^2^ = 46.3; k = 7).

**Figure 8:**
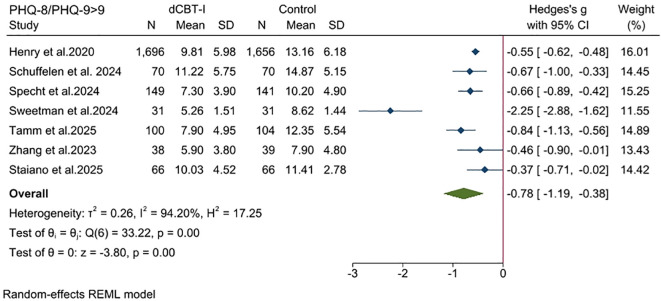
Subgroup analysis (Outcomes: PHQ-8 and PHQ-9).

Conduct a separate analysis of the 7 studies that used the PHQ-9/PHQ-8 as the outcome measure for depression and had a baseline value greater than 9 in the intervention group (50). The dCBT-I has shown moderate to large effects in moderate and severe depression (**Figure 8**; SMD = -0.78; 95% CI: -1.19, -0.38; *p* < 0.05; I^2^ = 94.2; k = 7).

### 3.6 Sensitivity analysis and publication bias

Sensitivity analyses were performed by removing two studies with insomnia whose outcomes were not ISI and one study in which the intervention was a self-developed app. After the exclusion of these studies, the therapeutic effect of dCBT-I was demonstrated for sleep (**Supplementary Figure 1**; SMD = -1.07; 95% CI: -1.51, -0.63; *p* < 0.05; I^2^ = 97.33; k = 14).

Visual inspection of funnel plots (**Supplementary Figure 5** for sleep outcome, **Supplementary Figure 6** for depression) and the Egger test was conducted under the random-effects model with the REML method, and the results showed no evidence of publication bias (*p* = 1.000).

## 4 Discussion

The current meta-analysis aims to evaluate the efficacy of dCBT-I without therapist guidance on insomnia and depression, and to examine whether there are differences in the efficacy among different forms of dCBT-I. By aggregating the data obtained from eligible RCTs, our results indicated that, compared with the control group, the intervention group was effective in alleviating insomnia, depressive symptoms, and the SE, TST, SOL, and WASO indicators in the sleep diary after unguided dCBT-I treatment. Among them, there is a large effect on insomnia symptoms and a medium effect on depressive symptoms. This is somewhat different from the results reported in the previous meta-analysis examining the impact of dCBT-I on depression, which indicated a mild to moderate effect on depression (30). However, previous studies were limited by the relatively small number of available studies and included other co-patient populations, which might have masked some of the effects of dCBT-I on depression. Due to an increase in sample size to 16 RCTS and the exclusion of the influence of other comorbidities, this updated meta-analysis further supports the effectiveness of self-service dCBT-I.

Although the pooled effect of dCBT-I on depressive symptoms was moderate, there was considerable heterogeneity in the magnitude of the observed effect. The diversity observed aligns with prior research, foreseeable due to the varied participant pool in these studies, the outcome metrics used, the types of CBT-I administered, and the initial severity of depression. Although dCBT-I was developed for the treatment of insomnia, current research results show that dCBT-I intervention is beneficial for reducing clinical depressive symptoms and can effectively complement therapy. In addition to alleviating depressive symptoms, the improvement in the severity of insomnia in this study was usually consistent with the improvement reported in previous meta-analysis reviews of the severity of insomnia in dCBT-I, SE, TST, SOL, and WASO (15).

Subgroup analyses revealed that both online-delivered and app-based dCBT-I demonstrated favorable outcomes on insomnia and depression, without notable distinctions in treatment efficacy between the two modalities. This similarity in effectiveness could be attributed to the convergence of online platforms and apps in user experience aspects, including interface design and interaction modalities, facilitated by advancements in mobile Internet technology. Consequently, the differences in user experience between different delivery media may be minimal, thereby exerting limited influence on treatment outcomes.

For patients with moderate and severe depression, dCBT-I shows moderate to large effects, approaching the “large effect” threshold. This differs from previous research on major depressive disorder, which found moderate effects (51). Patients with moderate and severe depression are often accompanied by chronic insomnia, and the two form a vicious cycle. The dCBT-I directly breaks this vicious cycle by improving insomnia: after sleep is restored, neurotransmitter secretion rhythms are stabilized, and the overactivation of the Hypothalamic axis is suppressed, which in turn alleviates the core symptoms of depression (e.g., lack of energy, low mood) (52). It contains cognitive reframing techniques that can correct the patient’s negative cognitions about sleep, and this thought adjustment will migrate to negative cognitions about depression; concurrently, while sleep restriction therapy may initially induce transient fatigue, it reduces emotional reactivity by enhancing sleep efficiency and restoring circadian rhythms and undermines the cognitive triad of depression; sleep restriction therapy indirectly increases patients’ daytime activity, which is often accompanied by behavioral withdrawal in patients with moderate-to-severe depression, and this passive activation breaks behavioral inhibition and enhances a sense of mastery, which in turn alleviates the core symptom of depression of helplessness. Given the significant heterogeneity between studies, further research is needed to validate these findings. Prior research suggests that therapist guided dCBT-I has superior outcomes in the treatment of depression compared to self-directed interventions (49). Future clinical trials could explore the effectiveness of virtual therapist interventions in this context.

This study is subject to certain limitations, such as small sample sizes potentially leading to inflated effect estimates and variations in control group compositions across studies, impacting result comparability. Subsequent research avenues may delve into the involvement of virtual therapists in dCBT-I, undertake extended follow-up assessments to gauge sustained efficacy, and juxtapose the efficacy of dCBT-I against CBT-I. Although statistical results indicate that dCBT-I demonstrates a relatively significant effect on both insomnia and depression, the extremely high heterogeneity renders the pooled estimates less stable. Digital interventions frequently encounter challenges related to low participant adherence. To enhance adherence, several studies have employed multiple strategies, such as personalized assessments and tailored treatment recommendations, incentive mechanisms, behavioral prompts for small daily actions, and fully automated reminder systems that deliver periodic prompts via push notifications or email. Furthermore, the vast majority of studies employ intention-to-treat (ITT) analysis to minimize bias arising from participant attrition, thereby ensuring robust results.

## 5 Conclusion

Unguided digital cognitive behavioral therapy for insomnia (dCBT-I) is an effective intervention for improving insomnia and depressive symptoms in adults with comorbid insomnia and depressive symptoms. The findings may not be generalizable to individuals at extremely high risk of suicide, those with severe mental comorbidities, or those with serious physical illnesses. The automated nature of dCBT-I not only overcomes geographical constraints but also diminishes treatment expenses, thereby affording patients improved treatment results.

## Data Availability

The original contributions presented in the study are included in the article/**Supplementary Material**. Further inquiries can be directed to the corresponding authors.
