# Supplementary material for: Effectiveness of unguided digital cognitive behavioral therapy for insomnia on depressive symptoms: a systematic review and meta-analysis of randomized controlled trials
Source: Front Psychiatry. 2026 Jan 12;16:1718949. doi: 10.3389/fpsyt.2025.1718949 (PMC12832971; doi:10.3389/fpsyt.2025.1718949)
Supplement: Supplementary file 1 [file Table1.docx]

Supplementary Material


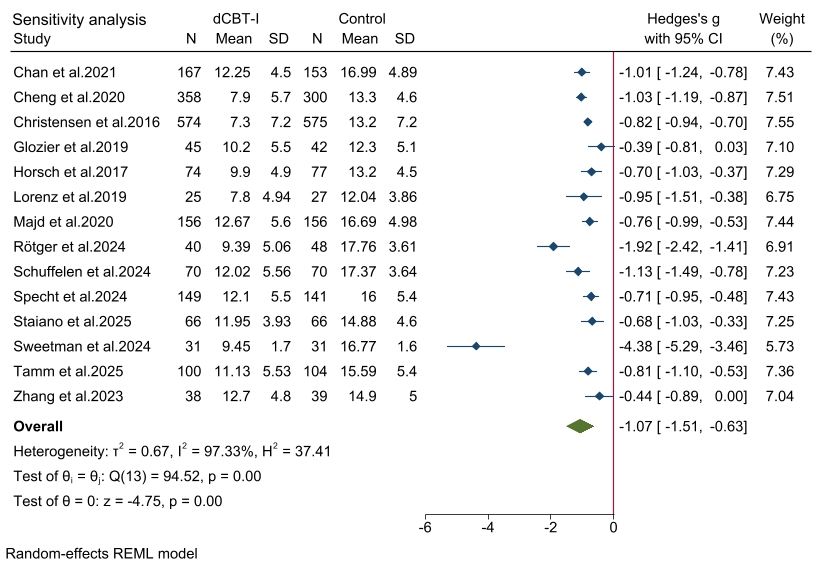
**Supplementary Fig. 1** Meta-analysis of the effect of dCBT-I on Sleep Outcome (ISI only)


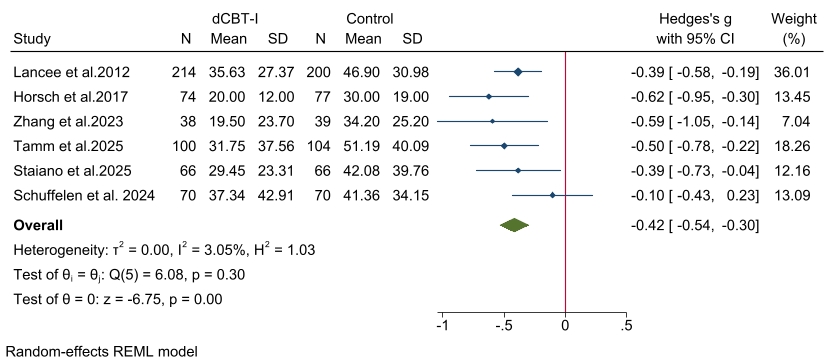


**Supplementary Fig. 2** Meta-analysis of the effect of dCBT-I on Sleep Diary Measures Sleep Onset Latency (SOL)


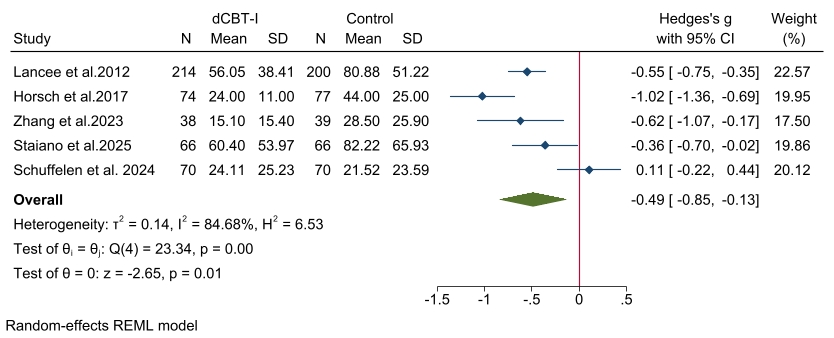


**Supplementary Fig. 3** Meta-analysis of the effect of dCBT-I on Sleep Diary Measures Wake After Sleep Onset (WASO)


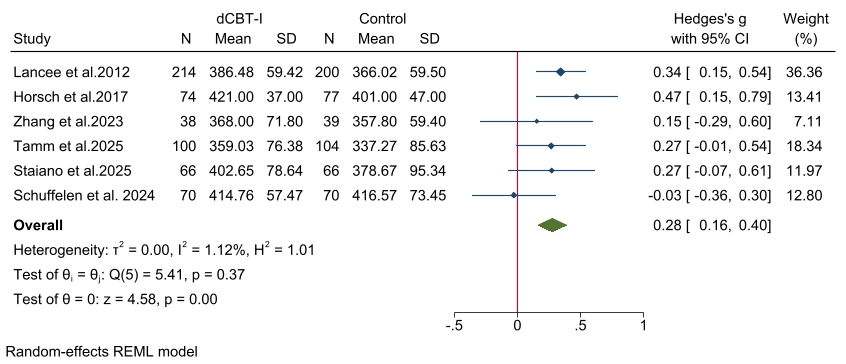


**Supplementary Fig. 4** Meta-analysis of the effect of dCBT-I on Sleep Diary Measures Total Sleep Time (TST)

**
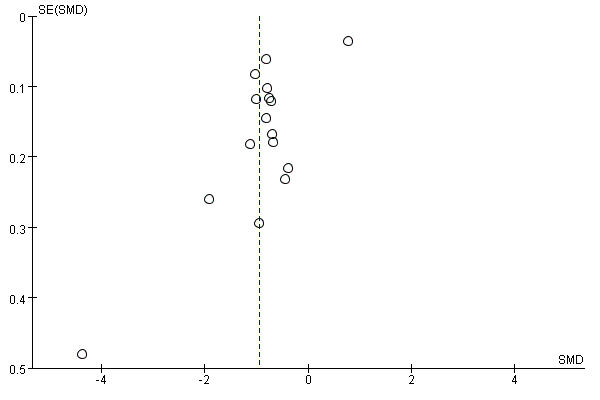
**

**Supplementary Fig. 5** The effect of dCBT-I on Sleep Outcome Funnel Plot


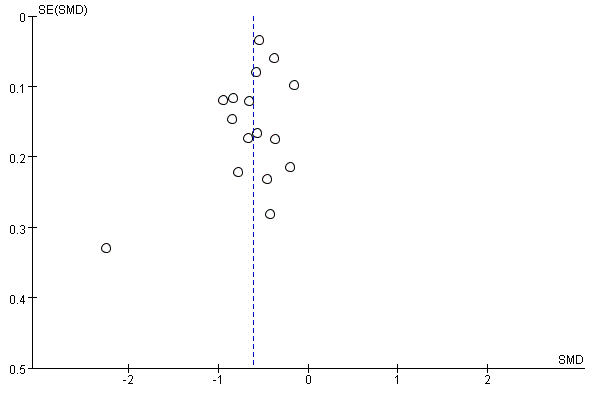


**Supplementary Fig. 6** The effect of dCBT-I on Depression Funnel Plot
